# A Novel β/ε Subunit Combination Expands the Tri-Subunit Acyl-CoA Carboxylase Repertoire in *Streptomyces coelicolor*

**DOI:** 10.3390/microorganisms14040733

**Published:** 2026-03-25

**Authors:** Shiyu Wu, Xue Yu, Yujie Wu, Xiaomin Niu, Ximing Chen, Tuo Chen, Wei Zhang, Guangxiu Liu, Paul Dyson

**Affiliations:** 1 State Key Laboratory of Cryospheric Science and Frozen Soil Engineering, Northwest Institute of Eco-Environment and Resources, Chinese Academy of Sciences, Lanzhou 730000, China; wushiyu23@mails.ucas.ac.cn; 2 Key Laboratory of Extreme Environmental Microbial Resources and Engineering of Gansu Province, Lanzhou 730000, China; yuxue@lzb.ac.cn (X.Y.); wuyujie18@mails.ucas.ac.cn (Y.W.); niuxiaomin22@mails.ucas.ac.cn (X.N.); ziaoshen@163.com (W.Z.); liugx@lzb.ac.cn (G.L.); 3 University of Chinese Academy of Sciences, Beijing 100049, China; 4 Key Laboratory of Ecological Safety and Sustainable Development in Arid Lands, Northwest Institute of Eco-Environment and Resources, Chinese Academy of Sciences, Lanzhou 730000, China; 5 Institute of Life Science, Swansea University Medical School, Singleton Park, Swansea SA2 8PP, UK; p.j.dyson@swansea.ac.uk

**Keywords:** acyl-CoA carboxylase, *Streptomyces coelicolor*, tri-subunit complex, β/ε subunits

## Abstract

Acyl-CoA carboxylase (YCC) complexes generate essential starter and extender units for fatty acid and polyketide biosynthesis in Actinobacteria. In *Streptomyces coelicolor*, two tri-subunit YCC complexes, acetyl-CoA carboxylase (ACC) and propionyl-CoA carboxylase (PCC), have been characterized. However, comparative genomic analyses indicate that β/ε subunits are more diversified than currently appreciated. Here, we identify a previously unrecognized β/ε pair, AccB2 and AccE2, and demonstrate that they assemble with the canonical α subunit to form a functional YCC complex. Both genes are transcribed in vivo, and co-immunoprecipitation (Co-IP) reveals association with AccA1 and AccA2, with AccE2 showing stronger relative association with AccA1-containing pull-downs. In vitro reconstitution confirms carboxylation activity toward acetyl-CoA, propionyl-CoA, and butyryl-CoA, which is strongly dependent on AccE2. These findings expand the YCC repertoire in *S. coelicolor* and support a modular assembly model in which alternative β/ε combinations contribute to functional diversification of YCC complexes.

## 1. Introduction

Biotin-dependent carboxylases are widely distributed in bacteria, archaea, and eukaryotes and play essential roles in fatty acid biosynthesis, polyketide production, and diverse carbon metabolic pathways [1,2]. Based on substrate specificity, this enzyme superfamily is classified into acyl-CoA carboxylases (YCC), pyruvate carboxylases (PC), and urea carboxylases (UC) [3]. Among them, YCC catalyzes the carboxylation of short-chain acyl-CoAs, generating key precursor molecules such as malonyl-CoA, methylmalonyl-CoA [3,4]. These metabolites are central intermediates in fatty acid and polyketide biosynthesis, positioning YCC as a critical node in carbon flux distribution between primary and secondary metabolism [5,6].

The YCC reaction is mediated by the coordinated action of three functional domains: biotin carboxylase (BC), biotin carboxyl carrier protein (BCCP), and carboxyltransferase (CT) [7,8,9]. The architectural organization of these domains varies substantially across different organisms. In *Escherichia coli*, acetyl-CoA carboxylase (ACC) consists of four separate subunits [10,11], whereas in eukaryotes, ACC is encoded as a single multidomain polypeptide [2]. In contrast, Actinobacteria such as *Streptomyces*, *Corynebacterium*, and *Mycobacterium* possess a distinct tri-subunit YCC complex composed of an α subunit containing the BC and BCCP domains, a β subunit harboring the CT domain, and a small ε subunit [12,13,14]. These components assemble into higher-order oligomeric structures, typically forming α_6_β_6_ or α_2_β_2_ complexes [3]. The ε subunit has been shown to play a crucial role in modulating catalytic efficiency and stabilizing complex assembly [15].

Recent studies in Actinobacteria have further highlighted the structural and functional diversity of tri-subunit YCC complexes, including structural analysis of the ACC β subunit in *Streptomyces* and functional characterization of ε-associated YCC systems in *Mycobacteria* [2,16,17,18]. In *Streptomyces coelicolor*, two tri-subunit YCC complexes have been characterized: ACC and propionyl-CoA carboxylase (PCC) [1,19]. Both complexes share the same α subunit encoded by either *accA1* or *accA2*, while substrate specificity is primarily determined by distinct β/ε combinations—AccB/AccE for ACC and PccB/PccE for PCC. Although both ACC and PCC exhibit a degree of substrate promiscuity, their substrate ranges differ: ACC catalyzes the carboxylation of acetyl-CoA, propionyl-CoA, and butyryl-CoA, whereas PCC primarily acts on propionyl-CoA and butyryl-CoA [1,8]. Previous studies have shown that β and ε subunit genes in *Streptomyces* are typically arranged in close genomic proximity, reflecting a conserved genetic linkage characteristic of tri-subunit YCC complexes [12]. In our prior comparative genomic analysis centered on the α subunit, we systematically surveyed α-, β-, and ε-encoding genes across several hundred *Streptomyces* genomes. This analysis revealed a pronounced asymmetry in subunit copy numbers: α-encoding genes were relatively conserved in number, whereas β and ε genes frequently exhibited multi-copy expansion. Available comparative genomic evidence suggests that β/ε subunits are more prone to duplication and diversification than the α subunit in *Streptomycetes*. This genomic organization suggests that, within a given organism, a limited number of α catalytic cores may associate with multiple alternative β/ε modules, potentially generating YCC complexes with distinct structural and functional properties. Such modular diversification raises the possibility that the YCC repertoire in *S. coelicolor* may extend beyond the two previously characterized complexes. Given this genomic asymmetry, it becomes important to determine whether additional β/ε combinations exist in the model organism *S. coelicolor*. Identifying and characterizing such combinations would refine our understanding of the YCC complement in this species and provide insight into how alternative β/ε modules contribute to carbon flux partitioning and metabolic specialization.

In this study, we conducted a comprehensive analysis of β and ε subunit families in the *S. coelicolor* genome and identified a previously uncharacterized β/ε pair, designated *accB2* and *accE2*. Through expression profiling, in vivo interaction assays, and in vitro enzymatic characterization, we evaluated their capacity to assemble with the canonical α subunit and assessed their catalytic properties. Our results demonstrate that AccB2 and AccE2 form a functional complex with the α subunit, representing a third tri-subunit YCC complex distinct from the previously characterized ACC and PCC complexes.

## 2. Materials and Methods

### 2.1. Strains, Plasmids, and General Techniques for DNA Manipulations

All bacterial strains and plasmids employed in this study are summarized in Table 1. *Escherichia coli* DH5α served as the routine host for plasmid construction and propagation. For conjugative transfer into *Streptomyces*, *E. coli* ET12567 [20] was used as the donor strain. *S. coelicolor* M145 was cultivated at 30 °C on MS agar or in R2YE medium as indicated. *E. coli* strains were cultured in Luria–Bertani (LB) medium at 37 °C. When required, antibiotics were supplemented at the following final concentrations: kanamycin (50 μg/mL), hygromycin B (50 μg/mL), and chloramphenicol (34 μg/mL).

### 2.2. RNA Extraction and Quantitative Real-Time PCR Analysis

Total RNA was isolated from *S. coelicolor* M145 using a commercial RNA extraction kit (Tsingke, Beijing, China) according to the manufacturer’s instructions. First-strand cDNA synthesis was performed with the SynScript^®^ III RT SuperMix for qPCR (Tsingke, Beijing, China). The resulting cDNA was diluted threefold before quantitative PCR analysis. Quantitative real-time PCR (qPCR) was carried out using ArtiCanCEO SYBR qPCR Mix (Tsingke, Beijing, China) on a real-time PCR detection system. The housekeeping gene *hrdB* was employed as the internal reference for normalization. Relative transcription levels were calculated using the 2^−ΔΔCt^ method. Primer sequences used in this study are listed in Table 2. The qPCR data are provided in the Supplementary Information Table S1.

### 2.3. Western Blot

Cells were harvested and resuspended in lysis buffer containing 20 mM Tris–HCl (pH 7.5), 150 mM NaCl, 1% Triton X-100, 10% glycerol, 5 mM EDTA, and 1 mM PMSF. Cell disruption was performed by sonication on ice, followed by centrifugation at 12,000 rpm for 20 min at 4 °C to remove cell debris. The protein concentration of the clarified supernatant was determined using a BCA protein assay kit (Solarbio, Beijing, China). Equal amounts of total protein (50 μg per sample) were resolved on 12% SDS–PAGE gels and electrotransferred onto PVDF membranes. Membranes were blocked with 5% non-fat milk for 2 h at room temperature and then incubated overnight at 4 °C with anti-HA primary antibody. After washing, membranes were incubated with HRP-conjugated goat anti-mouse secondary antibody for 2 h at room temperature. Immunoreactive bands were detected using an enhanced chemiluminescence (ECL) detection system (Servicebio, Wuhan, China). Band intensities were quantified using ImageJ (version 1.54) software, with GAPDH serving as the loading control [24].

### 2.4. Co-Immunoprecipitation and LC–MS/MS Analysis

Exponentially growing cultures were collected and lysed in Triton X-100–based buffer by sonication on ice. After centrifugation to remove insoluble material, protein concentrations in the clarified lysates were quantified using a BCA assay (Solarbio, Beijing, China). For co-immunoprecipitation (Co-IP), equal amounts of total protein were incubated with Flag-tag NanoAb magnetic beads or control nanobody-conjugated beads (NuoyiBio, Tianjin, China) according to the manufacturer’s protocol. After extensive washing, bound proteins were eluted. A portion of each sample was analyzed by SDS–PAGE followed by silver staining as described previously [25,26], while the remaining material was subjected to in-solution tryptic digestion at 37 °C for 16–18 h before LC–MS/MS analysis.

#### 2.4.1. Liquid Chromatography

Peptide mixtures were separated using a reversed-phase C18 analytical column (0.15 mm × 150 mm, Column Technology Inc., Lombard, IL, USA). The mobile phases consisted of solvent A (0.1% formic acid in water) and solvent B (0.1% formic acid in 84% acetonitrile). Peptides were eluted with a linear gradient from 4% to 50% B over 50 min, followed by an increase to 100% B from 50 to 54 min, and maintained at 100% B until 60 min. Before each run, the column was equilibrated with 95% solvent A. Samples were first loaded onto a ZORBAX 300SB-C18 peptide trap column (Agilent Technologies, Wilmington, DE, USA) before analytical separation.

#### 2.4.2. Mass Spectrometry

Eluted peptides were analyzed on a Q Exactive HF-X mass spectrometer (Thermo Fisher Scientific, Waltham, MA, USA) coupled to a capillary high-performance liquid chromatography system. Data were acquired in positive ion mode using a data-dependent acquisition strategy, in which each full MS scan was followed by MS/MS analysis of the ten most intense precursor ions. The total run time for each sample was 60 min.

#### 2.4.3. Data Analysis

Raw mass spectrometry data were processed using MaxQuant (version 1.5.5.1) against a custom protein database (Supplementary Information Table S2). Protein identification and label-free quantification were performed using default parameters unless otherwise specified.

### 2.5. Protein Expression and Purification

Empty pET-41a(+) vector and the corresponding YCC expression constructs were individually transformed into *E. coli* BL21(DE3). Transformants were cultured in LB medium supplemented with kanamycin (50 μg/mL) at 37 °C with shaking at 220 rpm. When the cultures reached an OD_600_ of approximately 0.6, flasks were placed on ice for 30 min before induction. Protein expression was induced by the addition of isopropyl β-D-1-thiogalactopyranoside (IPTG) to a final concentration of 0.25 mM, followed by incubation at 18 °C for 20 h with shaking. Cells were harvested by centrifugation at 5000 rpm for 10 min at 4 °C and resuspended in lysis buffer (40 mM Tris–HCl, pH 7.0, 200 mM NaCl, 10% glycerol) at a ratio of 1 g wet cell pellet per 10 mL buffer. Cell disruption was performed by sonication on ice. Insoluble material was removed by centrifugation at 10,000× *g* for 30 min at 4 °C, and the clarified supernatant was subjected to nickel-affinity chromatography for His-tagged protein purification. After loading, the column was washed with buffer containing 95 mM imidazole (40 mM Tris–HCl, pH 7.0, 200 mM NaCl, 10% glycerol) to remove nonspecifically bound proteins. Target proteins were eluted with a buffer containing 500 mM imidazole in the same base composition. Eluted fractions were desalted and concentrated using centrifugal ultrafiltration devices (Amicon, MilliporeSigma, Burlington, MA, USA). Finally, purified proteins were exchanged into storage buffer (50 mM HEPES, pH 7.0, 10% glycerol), aliquoted, and stored at −20 °C until further use.

### 2.6. Enzyme Assay

Enzymatic activity of the reconstituted YCC complexes was determined using a coupled spectrophotometric assay in which ADP production was linked to pyruvate kinase (PK) and lactate dehydrogenase (LDH), and NADH oxidation was monitored at 340 nm [27,28]. Reactions were carried out in a total volume of 100 μL containing 50 mM HEPES buffer (pH 7.5), 10 mM NaHCO_3_, 1.5 mM ATP, 0.4 mM NADH, 50 mM KCl, 10 mM MgCl_2_, 0.5 mM phosphoenolpyruvate (PEP), 3.5 U pyruvate kinase, 3.5 U lactate dehydrogenase, and 1 μM purified YCC complex. Reactions were initiated by the addition of acyl-CoA substrates, and the decrease in absorbance at 340 nm, corresponding to NADH oxidation, was recorded continuously for 15 min at 30 °C using a SpectraMax 190 microplate spectrophotometer (Molecular Devices, San Jose, CA, USA). Initial velocities were calculated from the linear portion of the reaction curves. For steady-state kinetic analysis, acetyl-CoA, propionyl-CoA, and butyryl-CoA were assayed at final concentrations of 0, 0.01, 0.1, 0.2, 0.5, and 1 mM. Kinetic parameters were obtained by fitting the data to the Michaelis–Menten equation using Origin software (version 2021). The kinetic parameters shown in Table 3 are presented as mean ± SD from independent experiments. Representative Michaelis–Menten plots are provided in Figure S1.

## 3. Results

### 3.1. Identification of an Additional β/ε Subunit Pair in S. coelicolor

To identify potential additional ε subunits associated with YCC complexes, we systematically surveyed ε subunit family proteins encoded in the *S. coelicolor* genome. In addition to the previously characterized AccE and PccE, we identified an additional gene, *sco6285*, encoding a protein belonging to the acyl-CoA carboxylase ε subunit family (Pfam: PF13822). This protein shared only ~30% amino acid sequence identity with the ACC-specific ε subunit AccE and the PCC-specific ε subunit PccE, indicating that it represents a divergent member of the ε subunit family. We therefore designated this gene *accE2*. Given the well-established genetic linkage between β and ε subunit genes in tri-subunit YCC complexes [1], we examined the genomic region surrounding *accE2*. Immediately upstream of *accE2*, we identified *sco6284*, encoding a predicted acyl-CoA carboxylase β subunit, which we designated *accB2*. Amino acid sequence analysis showed that AccB2 shares approximately 75% sequence identity with the ACC-specific β subunit AccB, consistent with its classification as a carboxyltransferase β subunit.

**Figure 1:**
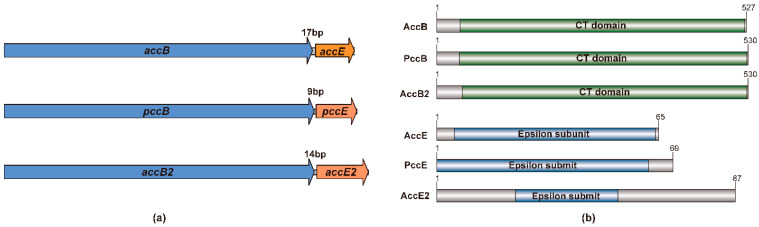
Domain organization and genomic arrangement of canonical and newly identified β/ε subunits in *S. coelicolor*. (**a**) Genomic organization of β and ε subunit-encoding genes in *S. coelicolor*. In all three systems, the β and ε genes are arranged in proximity and oriented in the same transcriptional direction. The intergenic distances are 17 bp for *accB*-*accE*, 9 bp for *pccB*-*pccE*, and 14 bp for the newly identified *accB2*-*accE2* pair, consistent with conserved genetic coupling of β/ε subunits in tri-subunit YCC complexes. (**b**) Schematic representation of the domain architectures of β and ε subunits from ACC, PCC, and the newly identified ACC2 complex. AccB, PccB, and AccB2 contain conserved carboxyltransferase (CT) domains of comparable length. AccE, PccE, and AccE2 all possess characteristic ε subunit domains, although AccE2 displays a distinct overall length relative to the canonical ε subunits. Colored regions indicate annotated conserved domains, whereas grey regions represent sequence regions outside the annotated domains. Amino acid lengths are indicated at the C-termini of each protein.

Genomic organization analysis revealed that *accB2* and *accE2* are located adjacent to each other on the chromosome and are transcribed in the same direction, separated by a 14 bp intergenic region. This arrangement suggests that the two genes may form a co-transcribed operon. For comparison, the intergenic distances between *accB* and *accE*, and between *pccB* and *pccE*, are 17 bp and 9 bp, respectively (Figure 1a).

Domain analysis further confirmed that AccB2 contains the conserved carboxyltransferase β subunit domain, whereas AccE2 harbors the characteristic ε subunit domain architecture. These features are consistent with those observed in the previously characterized AccB/PccB and AccE/PccE subunits (Figure 1b).

Collectively, the low sequence identity of AccE2 to canonical ε subunits, together with the conserved domain architecture and tight genomic linkage of *accB2* and *accE2*, supports the conclusion that they represent an additional β/ε subunit pair in *S. coelicolor* with the potential to assemble into a distinct YCC complex.

### 3.2. Expression Analysis of AccB2 and AccE2 in S. coelicolor

**Figure 2:**
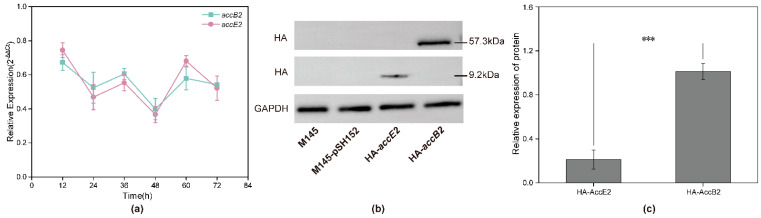
Transcriptional dynamics and protein expression of *accB2* and *accE2* in *S. coelicolor*. (**a**) Relative transcription levels of *accB2* and *accE2* at different time points (12, 24, 36, 48, 60, and 72 h) during growth. Transcript abundance was quantified by qPCR and normalized to the internal reference gene (*hrdB*). Data are presented as mean ± standard deviation (*n* = 3). Both genes were expressed throughout the time course and displayed similar temporal expression patterns. (**b**) Western blot (WB) analysis of AccB2 and AccE2 protein expression in *S. coelicolor*. HA-tagged AccB2 (~57.3 kDa) and AccE2 (~9.2 kDa) were detected using an anti-HA antibody in the corresponding recombinant strains. No specific bands were observed in the wild-type strain (M145) or empty vector control (M145-pSH152). GAPDH was used as a loading control. (**c**) Quantification of AccB2 and AccE2 protein levels normalized to GAPDH. Data are presented as mean ± standard deviation (*n* = 3). Statistical significance was determined by Student’s *t*-test (*** *p* < 0.001).

To assess the expression of *accB2* and *accE2* during growth, samples were collected at six time points (12, 24, 36, 48, 60, and 72 h) covering the growth cycle of strain M145. Relative transcript levels were quantified by qPCR using *hrdB* as the internal reference gene (Figure 2a). Both genes were detectably transcribed at all time points. Transcript abundance was relatively high at 12 h, decreased at 24 h, partially recovered at 36 h, reached a minimum at 48 h, and increased again at 60 h before stabilizing at 72 h. Across the entire time course, *accB2* and *accE2* exhibited highly similar transcriptional profiles, with synchronized fluctuations in transcript levels. The overall amplitude of transcriptional variation was moderate.

To verify protein expression, Western blot analysis was performed using samples collected during the exponential phase (Figure 2b). In strains expressing HA-tagged constructs, distinct bands corresponding to the predicted molecular weights of AccB2 (~57.3 kDa) and AccE2 (~9.2 kDa) were detected. No corresponding signals were observed in the wild-type or empty-vector controls. GAPDH was used as a loading control and showed consistent expression across all samples. Densitometric analysis indicated that, under identical detection conditions, the signal intensity of AccB2 was approximately 4.8-fold higher than that of AccE2, indicating differential protein accumulation between the two subunits (Figure 2c).

Collectively, these data demonstrate that *accB2* and *accE2* are expressed at both the transcript and protein levels in *S. coelicolor*.

### 3.3. AccB2 and AccE2 Associate with AccA1 and AccA2 In Vivo with Distinct Relative Association Patterns

To determine whether AccB2 and AccE2 are associated with the α subunit in vivo, we constructed strains expressing AccA1 or AccA2 fused to either N- or C-terminal Flag tags under their native promoters. Co-immunoprecipitation (Co-IP) was performed during exponential growth, and precipitated proteins were identified by LC–MS/MS. Label-free quantification (LFQ) intensities were log_10_-transformed before comparative and statistical analyses.

**Figure 3:**
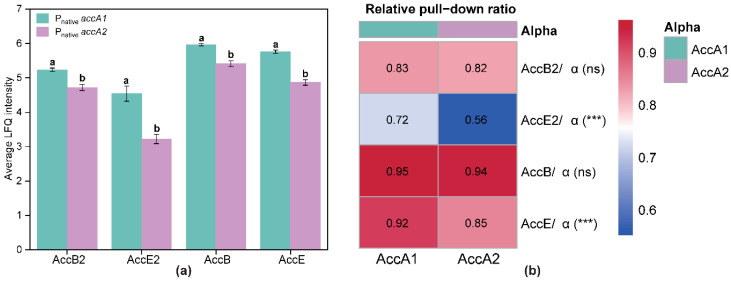
In vivo association of AccB2 and AccE2 with AccA1 and AccA2 revealed by co-immunoprecipitation (Co-IP) and relative assembly analysis. (**a**) Average log_10_ LFQ intensities of β and ε subunits detected in AccA1- and AccA2-based pull-down samples. Bars represent mean ± SD from independent experiments. AccB2, AccE2, AccB, and AccE were consistently detected in both α backgrounds. For all detected subunits, signal intensities were significantly higher in the AccA1 pull-down samples than in the AccA2 samples. Different lowercase letters indicate statistically significant differences (Welch’s *t*-test, *p* < 0.001). (**b**) Relative pull-down ratios (protein/α) showing normalized association levels of β and ε subunits with AccA1 or AccA2. LFQ intensities were normalized to the corresponding α subunit signal to account for differences in bait enrichment. AccB2/α exhibited comparable ratios in both α backgrounds (ns), whereas AccE2/α showed a significantly higher relative association with AccA1 than with AccA2 (***). The canonical ACC subunits AccB/α and AccE/α are shown for comparison. Welch’s *t*-test evaluated statistical significance; ns, not significant; ***, *p* < 0.001.

We first evaluated whether the Flag tag position affected Co-IP results. In both AccA1 and AccA2 backgrounds, the LFQ signal patterns of AccB2, AccE2, and the canonical ACC subunits AccB and AccE were comparable between N- and C-terminal tagging, with no systematic differences observed (Supplementary Information Table S2). These data indicate that tag placement did not measurably influence protein co-recovery or pull-down efficiency. Accordingly, data from N- and C-terminally tagged strains were combined for subsequent analyses. The averaged log_10_ LFQ intensities are shown in Figure 3a.

Both AccB2 and AccE2 were consistently detected in AccA1 and AccA2 pull-down samples, indicating that each subunit can be recovered in association with either α isoform in vivo. The canonical ACC subunits AccB and AccE were also robustly enriched under both conditions, supporting the reliability of the Co-IP assay. Statistical analysis using Welch’s *t*-test revealed that LFQ intensities for all four detected proteins were significantly higher in AccA1 pull-down samples compared with AccA2 samples (*p* < 0.001). Among these, AccE2 exhibited the largest difference between the two α backgrounds, suggesting isoform-dependent association behavior.

Because absolute LFQ intensities may be influenced by differences in bait enrichment, we normalized the LFQ signal of each β/ε subunit to the corresponding α subunit signal (protein/α) to estimate relative association levels. These ratios were visualized as a heatmap (Figure 3b). For the canonical ACC subunits, AccB/α and AccE/α ratios remained high (approximately 0.9) in both α backgrounds. AccB/α showed no significant difference between AccA1 and AccA2 (ns), indicating comparable association with both α isoforms under the Co-IP conditions. AccB2/α displayed a similar pattern, with comparable ratios in AccA1 and AccA2 backgrounds (approximately 0.82–0.83, ns), suggesting similar relative association of the β subunit in the two pull-down backgrounds. In contrast, ε subunits exhibited more pronounced α-dependent variation. The AccE/α ratio was modestly reduced in the AccA2 background, becoming statistically significant. More strikingly, the AccE2/α ratio differed substantially between the two α backgrounds, with values of approximately 0.73 in the AccA1 pull-down background and 0.56 in the AccA2 pull-down background. These results indicate that although AccE2 associates with both α isoforms in vivo, its relative incorporation efficiency differs between AccA1- and AccA2-containing complexes.

Overall, these findings indicate that AccB2 and AccE2 are associated with α-containing protein assemblies in vivo. Still, their relative association patterns are not identical: AccB2 is recovered at similar levels with both α isoforms, whereas AccE2 shows a stronger relative association with AccA1-containing pull-downs.

### 3.4. In Vitro Enzymatic Characterization of a YCC Complex Assembled with AccB2 and AccE2

To determine whether AccB2 and AccE2 assemble with the α subunit to form a catalytically active YCC complex, we co-expressed the corresponding recombinant proteins in *E. coli*. In parallel, the canonical ACC (AccA + AccB + AccE) and PCC (AccA + PccB + PccE) complexes were reconstituted as reference systems. The purified complexes were then subjected to in vitro kinetic analysis. Because recombinant expression in *E. coli* does not recapitulate the native regulatory context of *S. coelicolor*, and because AccA1 and AccA2 encode identical α-subunit polypeptides, we did not observe significant differences in catalytic activity between AccA1- and AccA2-containing complexes under in vitro conditions. We therefore combined kinetic data from the two α backgrounds for subsequent analysis.

As expected, the canonical ACC complex catalyzed the carboxylation of acetyl-CoA, propionyl-CoA, and butyryl-CoA, whereas the PCC complex displayed activity primarily toward propionyl-CoA and butyryl-CoA (Table 3 and Figure S1). These substrate profiles are consistent with previous reports and confirm the validity of our reconstitution system [1,8,19]. We next examined the newly identified complex composed of AccB2 and AccE2 (hereafter referred to as ACC2). ACC2 exhibited detectable carboxylation activity toward all three substrates tested. The catalytic efficiencies (kcat/Km) were 1.99 ± 0.33 × 10^5^ M^−1^·min^−1^ for acetyl-CoA, 2.03 ± 0.31 × 10^5^ M^−1^·min^−1^ for propionyl-CoA, and 2.03 ± 0.48 × 10^5^ M^−1^·min^−1^ for butyryl-CoA. Although these values were lower than those of the canonical ACC complex, they remained within the 10^5^ M^−1^·min^−1^ range, demonstrating that ACC2 possesses intrinsic carboxylase activity. Given the established role of ε subunits in modulating catalytic performance in tri-subunit YCC complexes [1], we further assessed the activity of an ACC2 complex assembled in the absence of AccE2. Removal of AccE2 resulted in a pronounced reduction in catalytic efficiency. The kcat/Km values decreased to 0.60 ± 0.18 × 10^5^ M^−1^·min^−1^ for acetyl-CoA, 0.18 ± 0.05 × 10^5^ M^−1^·min^−1^ for propionyl-CoA, and 0.10 ± 0.03 × 10^5^ M^−1^·min^−1^ for butyryl-CoA. For propionyl-CoA and butyryl-CoA, the reduction exceeded one order of magnitude. These results demonstrate that AccE2 substantially enhances the catalytic performance of the ACC2 complex.

Taken together with the transcriptional, protein expression, and Co-IP data, these findings indicate that AccB2 and AccE2 are associated with the α subunit in vivo and can form a catalytically competent carboxylase complex under in vitro reconstitution conditions. The strong dependence of enzymatic activity on the ε subunit further supports the classification of AccB2-AccE2 as components of a previously unrecognized tri-subunit YCC complex in *S. coelicolor*.

## 4. Discussion

In this study, we identified and functionally characterized a previously unrecognized β/ε subunit pair, AccB2 and AccE2, in *S. coelicolor*. We show that these subunits can assemble with the canonical α subunit to form a catalytically competent tri-subunit YCC complex. Whereas previous work in Actinobacteria has largely focused on the canonical ACC and PCC complexes, our findings provide evidence for an additional β/ε combination in *S. coelicolor*, thereby expanding the structural and functional scope of the YCC system in this model organism.

A defining feature of tri-subunit YCC complexes in Actinobacteria is the presence of the small ε subunit and its strong influence on catalytic performance. Earlier studies in *S. coelicolor* and related species have shown that ε subunits not only enhance catalytic efficiency but also play a critical role in complex assembly and stability [1,18]. In Mycobacteria and other Actinobacteria, the ε-encoding gene is typically located immediately downstream of the β subunit gene, reflecting conserved genetic organization and functional coupling [1]. In the present study, *accB2* and *accE2* exhibit a similar genomic arrangement, and removal of AccE2 results in a marked reduction in catalytic efficiency in vitro. These observations reinforce the structural and functional importance of ε subunits within tri-subunit YCC systems.

With respect to substrate utilization, the ACC2 complex exhibits comparable catalytic efficiencies toward acetyl-CoA, propionyl-CoA, and butyryl-CoA. This relatively balanced substrate profile differs from the more pronounced substrate preferences reported for canonical ACC and PCC complexes [1,12,29]. Substrate promiscuity is a recurring feature among biotin-dependent carboxylases and has been proposed to enhance metabolic flexibility [30,31,32]. In this context, the evenly distributed catalytic efficiency of ACC2 suggests a role distinct from that of a primary flux-dominant enzyme. Rather than serving as a dedicated route for a specific extender unit, ACC2 may operate under conditions requiring flexible redistribution of carbon flux.

The overall catalytic efficiency of ACC2 is lower than that of the canonical ACC complex. Deletion of *accB* has been reported to be lethal in *Streptomycetes*, underscoring the essential role of ACC in fatty acid biosynthesis and primary metabolism [12]. Although ACC2 catalyzes acetyl-CoA carboxylation in vitro, its reduced efficiency makes it unlikely to replace the canonical ACC complex as the principal source of malonyl-CoA under standard growth conditions. Instead, ACC2 may function in a context-dependent manner, contributing during specific growth phases or participating in fine-tuning precursor supply during metabolic transitions. In this regard, the AccB2/AccE2 combination appears to define a functional module distinct from the canonical AccB/AccE and PccB/PccE pairs. Compared with the known ACC and PCC complexes, this alternative β/ε pairing is associated with a more balanced substrate profile and lower overall catalytic efficiency, suggesting that variation in β/ε composition may expand YCC functional diversity beyond simple structural assembly.

Expression data provide additional support for this interpretation. Both *accB2* and *accE2* are transcribed throughout growth, although transcript levels fluctuate across developmental stages. Notably, our Co-IP data reveal that AccE2 displays a higher relative association with AccA1 than with AccA2. In previous work, we demonstrated functional differentiation between the two α isoforms, with AccA1 more closely linked to secondary metabolism and AccA2 primarily associated with primary metabolic processes [5]. This observation is therefore compatible with the possibility that ACC2 may participate in metabolic networks connected to secondary metabolite biosynthesis. At the same time, because Co-IP indicates protein association in vivo but does not by itself demonstrate direct binding or stable stoichiometric complex formation, this inference remains provisional. Thus, our data support a working model in which diversification of β/ε modules may enable functional specialization without duplication of the α catalytic core, although the precise physiological role of ACC2 will require direct in vivo validation.

**Figure 4:**
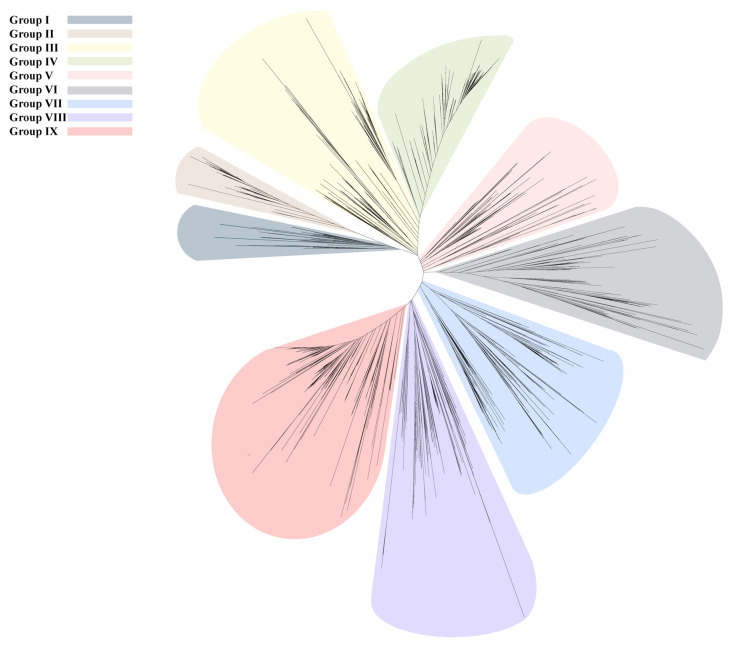
Phylogenetic analysis of actinobacterial ε-subunit homologs. ε-subunit homologs were identified using the PF13822 HMM profile, and 1726 curated actinobacterial sequences were retained after filtering based on species annotation and genetic linkage to β-subunit genes. Multiple sequence alignment was performed using MAFFT, and the phylogenetic tree was reconstructed using FastTree. Based on phylogenetic relationships and sequence similarity (>50%), the sequences were classified into 10 groups. The canonical AccE and PccE proteins are located in Group IV and Group IX, respectively, whereas AccE2 is located in Group VI.

From an evolutionary perspective, AccE2 exhibits limited sequence similarity to the canonical ACC- and PCC-type ε subunits. To further examine its evolutionary placement, ε-subunit homologs were identified using the PF13822 HMM profile from the UniProt database. Among the retrieved sequences, 1726 curated actinobacterial ε-subunit homologs were retained for phylogenetic analysis after filtering based on defined species annotation and genetic linkage to β-subunit genes. Multiple sequence alignment was performed using MAFFT (version 7.526), and the phylogenetic tree was reconstructed using FastTree (version 2.2) (Figure 4). Branch support values were calculated and are shown for selected nodes in the enlarged Group VI subtree (Figure S1). This analysis showed that the canonical AccE and PccE proteins are located in Group IV and Group IX, respectively, whereas AccE2 is positioned in Group VI, outside the canonical AccE and PccE clades, and groups instead with ε-like proteins from genera such as *Rhodococcus* and *Corynebacterium* (Figure 4 and Figure S1). These results suggest that AccE2 represents a differentiated ε-subunit lineage within Actinobacteria. This phylogenetic separation supports the notion that diversification of ε subunits may constitute a key evolutionary mechanism underlying functional expansion within YCC systems. Comparative genomic analyses further reinforce this view; whereas α subunit gene numbers remain relatively conserved across *Streptomycetes*, β and ε subunits frequently undergo duplication and diversification [14,18,33,34]. Such an organizational pattern is consistent with a model in which a conserved α catalytic scaffold is combined with alternative β/ε modules, thereby generating YCC complexes with distinct assembly preferences and catalytic characteristics.

An additional observation deserves consideration. Although no difference in catalytic efficiency was observed between AccA1- and AccA2-containing complexes under in vitro conditions, Co-IP experiments revealed clear differences in relative association patterns in vivo. This contrast likely reflects the influence of the cellular environment. Within the cell, complex formation may be shaped by spatial organization, local metabolite concentrations, interactions with other proteins, or post-translational modifications—factors not captured in simplified in vitro assays [35,36,37]. Determining whether ACC2 preferentially contributes to specific metabolic pathways in vivo will require further investigation, including genetic disruption, in vivo activity measurements, and metabolomic or flux-based analyses.

In summary, our findings expand the current understanding of YCC organization in *S. coelicolor* by identifying a novel β/ε subunit combination capable of forming a functional carboxylase complex. ACC2 exhibits distinct assembly characteristics and catalytic features relative to canonical ACC and PCC complexes and may represent an auxiliary carboxylase that could contribute to modulating carbon flux. These results provide new insight into how diversification of β/ε subunits contributes to metabolic specialization and evolutionary adaptation within actinobacterial YCC systems.

## Figures and Tables

**Table 1:** Strains and plasmids used in this study.

Strains and Plasmids	Genotype	Reference
**Strains**		
*S. coelicolor* strains		
M145	The parental strain	Bentley et al. [21]
M145/pSH152	M145 containing the plasmid pSH152	This study
M145/HA-*accB2*	M145 carrying *accB2* under its native promoter with an N-terminal HA tag	This study
M145/HA-*accE2*	M145 carrying *accE2* under its native promoter with an N-terminal HA tag	This study
M145/Flag-*accA1*	M145 carrying *accA1* under its native promoter with an N-terminal Flag tag	This study
M145/*accA1*-Flag	M145 carrying *accA1* under its native promoter with a C-terminal Flag tag	This study
M145/Flag-*accA2*	M145 carrying *accA2* under its native promoter with an N-terminal Flag tag	This study
M145/*accA2*-Flag	M145 carrying *accA2* under its native promoter with a C-terminal Flag tag	This study
*E. coli* strains		
DH5α	F-*φ80lacZΔM15Δ(lacZYA-argF)U169recA1endA1hsdR17(rk-mk+)phoAsupE44λ-thi-1gyrA96relA1*	TransGen Biotech Co., Ltd. (Beijing, China).
ET12567/pUZ8002	*dam-13*::Tn9 *dcm-6 hsdM*; containing the non-transmissible RP4 derivative plasmid pUZ8002	Flett et al. [20]
**Plasmids**		
pSH152	*E. coli-S. coelicolor* shuttle vector, hygromycin resistance	Mistry et al. [22]
pSH152-1	pSH152 carrying N-terminal HA-tagged *accB2* driven by its native promoter	This study
pSH152-2	pSH152 carrying N-terminal HA-tagged *accE2* driven by its native promoter	This study
pSH152-3	pSH152 carrying N-terminal Flag-tagged *accA1* driven by its native promoter.	This study
pSH152-4	pSH152 carrying C-terminal Flag-tagged *accA1* driven by its native promoter.	This study
pSH152-5	pSH152 carrying N-terminal Flag-tagged *accA2* driven by its native promoter.	This study
pSH152-6	pSH152 carrying C-terminal Flag-tagged *accA2* driven by its native promoter.	This study
pET41a	Kana ^r^, pBR322 origin, PT7	This study [23]
pET41a-*accA1*	Derivative pET41a plasmid containing intact *accA1*	This study
pET41a-*accA2*	Derivative pET41a plasmid containing intact *accA2*	This study
pET41a-*accB*	Derivative pET41a plasmid containing intact *accB*	This study
pET41a-*accE*	Derivative pET41a plasmid containing intact *accE*	This study
pET41a-*pccB*	Derivative pET41a plasmid containing intact *pccB*	This study
pET41a-*pccE*	Derivative pET41a plasmid containing intact *pccE*	This study
pET41a-*accB2*	Derivative pET41a plasmid containing intact *accB2*	This study
pET41a-*accE2*	Derivative pET41a plasmid containing intact *accE2*	This study

Kana ^r^, kanamycin resistance.

**Table 2:** Primers used for this study.

Primer	Sequence (5′-3′)
*accB2*-F	CGTACATCGTGATGGACTCCC
*accB2*-R	ATGAGCTCGTCGCGGTATTC
*accE2*-F	GCGAACCCCATGTCCGAGA
*accE2*-R	CATGGCCGCGGACAGGAC
*hrdB*-F	GAAGCTGACCAGATTCCGGC
*hrdB*-R	TTCGCTGCGACGCTCTTTC

**Table 3:** In vitro steady-state kinetic parameters of reconstituted YCC complexes.

		ACC	PCC	ACC2	ACC2(-AccE2)
**Acetyl-CoA**	**Km (μM)**	**93.24 ± 18.45**	**—**	**117.96 ± 14.10**	**87.41 ± 9.25**
	**kcat (min^−1^)**	**40.46 ± 3.30**	**—**	**23.48 ± 2.71**	**5.25 ± 1.46**
	**kcat/Km (×10^5^ M^−1^ min^−1^)**	**4.34 ± 0.93**	**—**	**1.99 ± 0.33**	**0.60 ± 0.18**
**Propionyl-CoA**	**Km (μM)**	**85.09 ± 13.12**	**72.28 ± 3.77**	**125.16 ± 11.45**	**131.87 ± 19.01**
	**kcat (min^−1^)**	**64.19 ± 1.55**	**110.26 ± 5.70**	**25.36 ± 3.18**	**2.34 ± 0.58**
	**kcat/Km (×10^5^ M^−1^ min^−1^)**	**7.54 ± 1.18**	**15.25 ± 1.11**	**2.03 ± 0.31**	**0.18 ± 0.05**
**Butyryl-CoA**	**Km (μM)**	**95.46 ± 24.03**	**98.66 ± 16.12**	**135.15 ± 29.56**	**124.52 ± 23.43**
	**kcat (min^−1^)**	**42.86 ± 2.32**	**65.44 ± 3.20**	**27.41 ± 2.54**	**1.25 ± 0.35**
	**kcat/Km (×10^5^ M^−1^ min^−1^)**	**4.49 ± 1.15**	**6.63 ± 1.13**	**2.03 ± 0.48**	**0.10 ± 0.03**

Values are presented as mean ± SD from three independent experiments. Initial velocities were measured at acyl-CoA concentrations of 0, 0.01, 0.1, 0.2, 0.5, and 1 mM and fitted to the Michaelis–Menten equation. —, no measurable activity detected under the assay conditions.

## Data Availability

The original contributions presented in this study are included in the article/Supplementary Material. Further inquiries can be directed to the corresponding authors.

## Supplementary Materials

The following supporting information can be downloaded at: https://www.mdpi.com/article/10.3390/microorganisms14040733/s1, Table S1: Quantitative real-time PCR (qPCR) data; Table S2: Log_10_-transformed LFQ intensities of co-immunoprecipitated proteins identified by LC–MS/MS; Figure S1: Representative Michaelis–Menten plots for reconstituted YCC complexes; Figure S2: Enlarged phylogenetic subtree of Group VI ε-subunit homologs showing the position of AccE2.
