# Supplementary material for: A Novel β/ε Subunit Combination Expands the Tri-Subunit Acyl-CoA Carboxylase Repertoire in Streptomyces coelicolor
Source: Microorganisms. 2026 Mar 25;14(4):733. doi: 10.3390/microorganisms14040733 (PMC13118682; doi:10.3390/microorganisms14040733)
Supplement: Supplementary file 1 [file microorganisms-14-00733-s001.zip › microorganisms-4223054-supplementary.pdf]

Table S1. Quantitative real-time PCR (qPCR) data

Sample Name	Target Name	CT	Ct Mean	Ct SD
M145 12H -1	*accB2*	23.970	23.985	0.014
M145 12H -2	*accB2*	23.816	23.821	0.046
M145 12H -3	*accB2*	24.051	24.077	0.024
M145 24H -1	*accB2*	23.031	22.998	0.029
M145 24H -2	*accB2*	22.992	22.942	0.052
M145 24H -3	*accB2*	22.648	22.660	0.010
M145 36H -1	*accB2*	22.913	22.888	0.022
M145 36H -2	*accB2*	23.077	23.023	0.051
M145 36H -3	*accB2*	22.784	22.809	0.061
M145 48H -1	*accB2*	22.729	22.691	0.043
M145 48H -2	*accB2*	22.710	22.726	0.023
M145 48H -3	*accB2*	22.830	22.836	0.090
M145 60H -1	*accB2*	22.886	22.843	0.037
M145 60H -2	*accB2*	22.675	22.683	0.008
M145 60H -3	*accB2*	22.819	22.771	0.069
M145 72H -1	*accB2*	24.025	23.972	0.048
M145 72H -2	*accB2*	24.075	24.054	0.026
M145 72H -3	*accB2*	24.178	24.159	0.063
M145 12H -1	*accE2*	23.870	23.858	0.012
M145 12H -2	*accE2*	23.676	23.755	0.132
M145 12H -3	*accE2*	23.821	23.826	0.006
M145 24H -1	*accE2*	23.185	23.180	0.050
M145 24H -2	*accE2*	23.087	23.077	0.064
M145 24H -3	*accE2*	22.899	22.813	0.083
M145 36H -1	*accE2*	23.181	23.102	0.075
M145 36H -2	*accE2*	22.918	22.928	0.008
M145 36H -3	*accE2*	23.028	23.089	0.083
M145 48H -1	*accE2*	22.774	22.744	0.049
M145 48H -2	*accE2*	23.025	23.020	0.040
M145 48H -3	*accE2*	22.793	22.845	0.050
M145 60H -1	*accE2*	22.461	22.457	0.016
M145 60H -2	*accE2*	22.521	22.658	0.131
M145 60H -3	*accE2*	22.444	22.467	0.049
M145 72H -1	*accE2*	23.973	23.964	0.027
M145 72H -2	*accE2*	24.262	24.321	0.064
M145 72H -3	*accE2*	24.054	24.102	0.043
M145 12H -1	*hrdB*	19.621	19.640	0.039
M145 12H -2	*hrdB*	19.610	19.654	0.089
M145 12H -3	*hrdB*	19.702	19.770	0.058
M145 24H -1	*hrdB*	18.592	18.592	0.009
M145 24H -2	*hrdB*	18.179	18.031	0.140

Table S1. Quantitative real-time PCR (qPCR) data (continued)

Sample Name	Target Name	CT	Ct Mean	Ct SD
M145 24H -3	*hrdB*	17.998	18.046	0.096
M145 36H -1	*hrdB*	18.511	18.506	0.060
M145 36H -2	*hrdB*	18.418	18.508	0.118
M145 36H -3	*hrdB*	18.548	18.440	0.099
M145 48H -1	*hrdB*	17.782	17.787	0.035
M145 48H -2	*hrdB*	17.702	17.837	0.229
M145 48H -3	*hrdB*	17.643	17.528	0.114
M145 60H -1	*hrdB*	18.141	18.177	0.176
M145 60H -2	*hrdB*	18.221	18.355	0.179
M145 60H -3	*hrdB*	18.240	18.289	0.071
M145 72H -1	*hrdB*	19.386	19.409	0.060
M145 72H -2	*hrdB*	19.532	19.437	0.084
M145 72H -3	*hrdB*	19.582	19.600	0.060

Table S2. Log_{10}-transformed LFQ intensities of co-immunoprecipitated proteins identified by LC–MS/MS

	Name	AccA1/AccA2	accB2	accE2	AccB	AccE
Pnative *accA1*_N	Intensity 1	6.27793	5.090998	4.009493	5.975547	5.770926
	Intensity 2	6.362596	5.289054	4.519277	6.033952	5.906664
	Intensity 3	6.283725	5.303423	4.626186	5.940586	5.715088
Pnative *accA1*_C	Intensity 4	6.237745	5.308233	4.859805	5.865015	5.786217
	Intensity 5	6.277669	5.241596	4.63483	5.965395	5.870511
	Intensity 6	6.299329	5.1825	4.589682	5.98063	5.523072
Pnative *accA2*_N	Intensity 7	5.658202	4.742961	3.010724	5.325269	4.807102
	Intensity 8	5.704939	4.607294	3.308564	5.447352	4.900995
	Intensity 9	5.647344	4.790004	3.361728	5.265396	4.726523
Pnative *accA2*_C	Intensity 10	5.755063	4.53362	3.187521	5.514448	4.743259
	Intensity 11	5.915363	4.777209	3.08636	5.608333	5.10804
	Intensity 12	5.742622	4.872226	3.380211	5.301119	4.916927

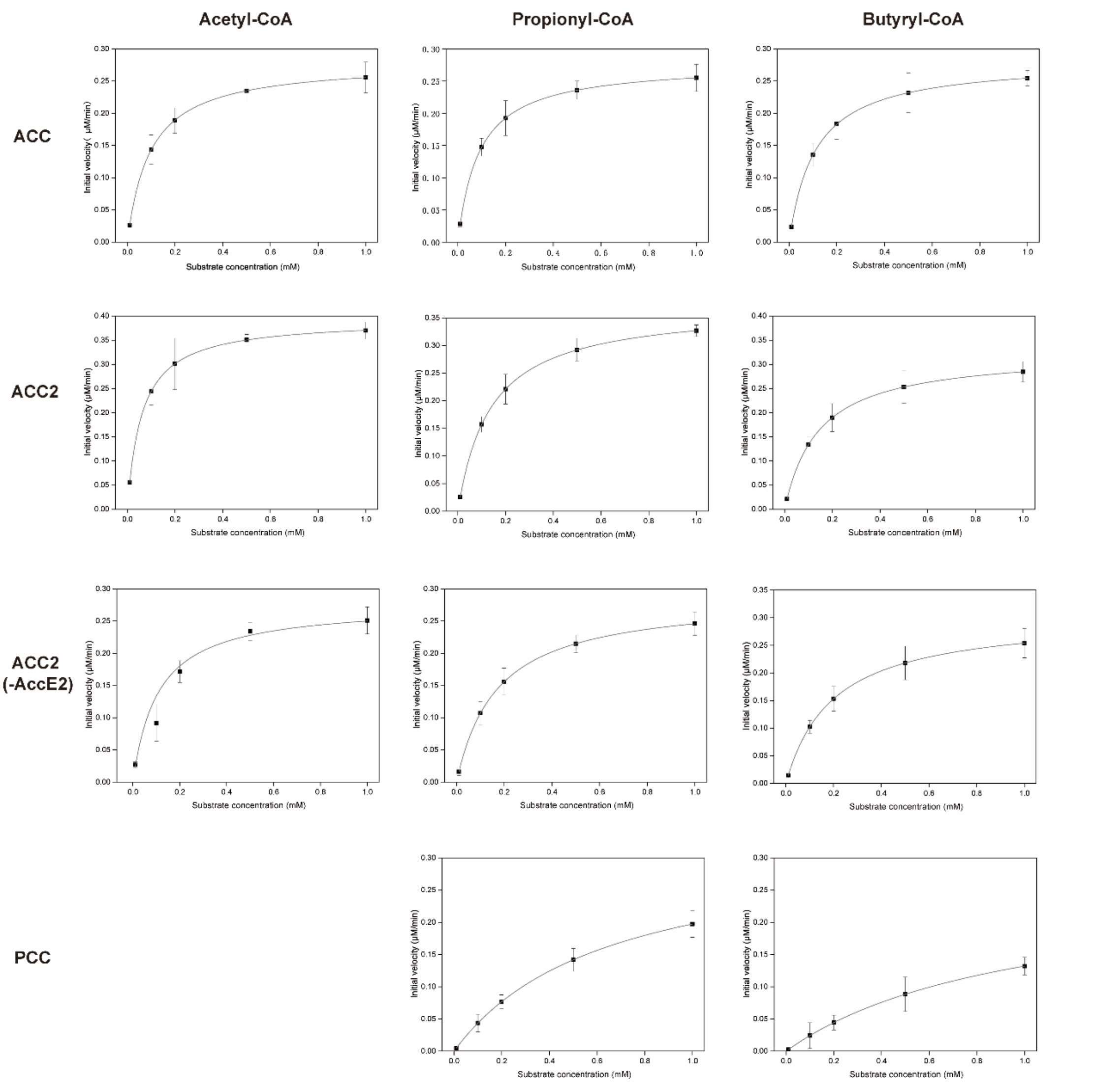

Figure S1. Representative Michaelis-Menten plots for reconstituted YCC complexes. Initial velocities were measured using acetyl-CoA, propionyl-CoA, or butyryl-CoA as substrates at final concentrations of 0, 0.01, 0.1, 0.2, 0.5, and 1 mM. Data points represent mean ± SD from independent experiments, and the curves show fits to the Michaelis-Menten equation generated in Origin. The corresponding kinetic parameters are summarized in Table 3.

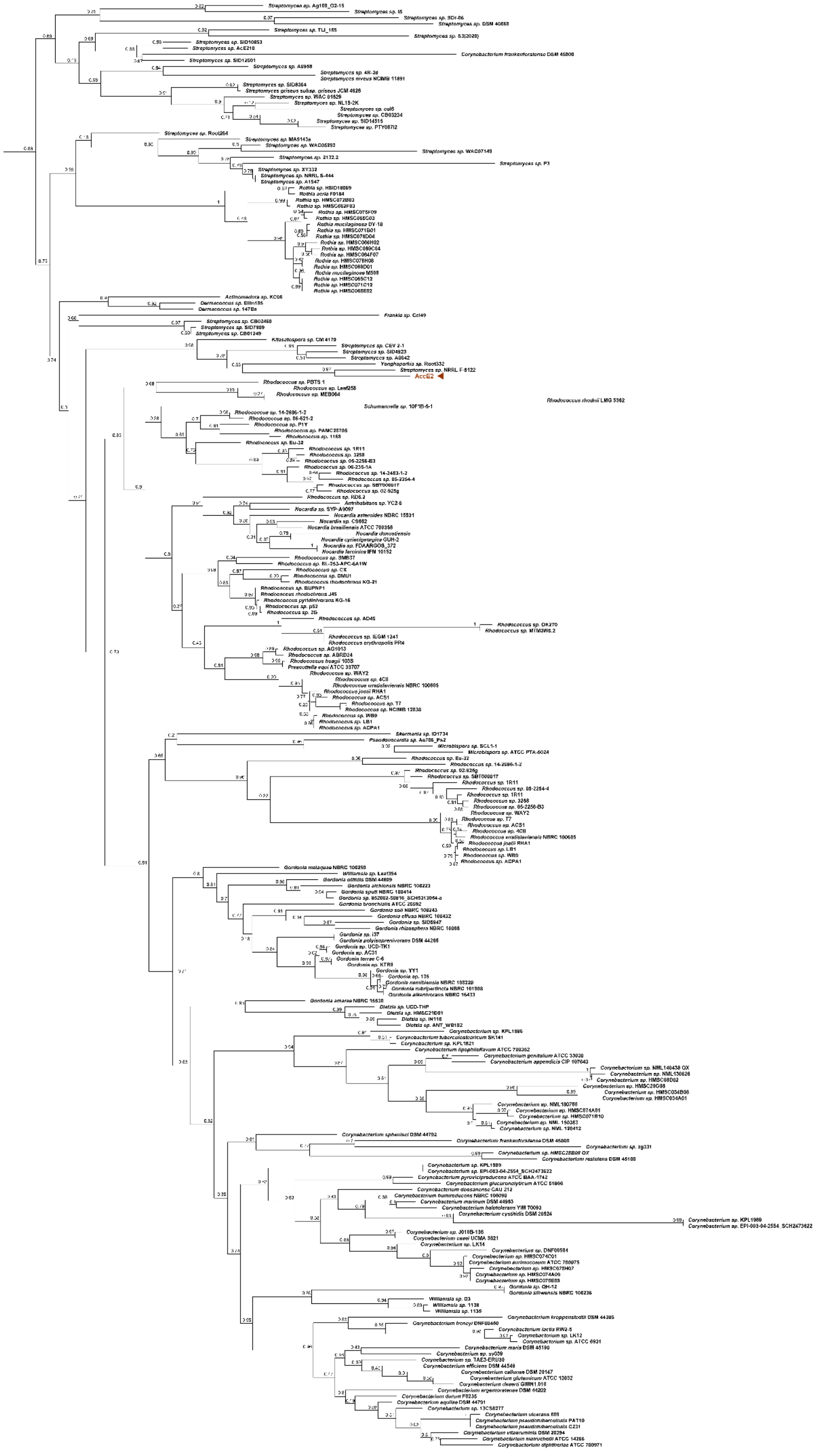

Streptomyces sp. Ag109_G2-15
Streptomyces sp. I5
Streptomyces sp. SDr-06
Streptomyces sp. DSM 40868
Streptomyces sp. TLI_185
Streptomyces sp. 53(2020)
Streptomyces sp. SID10853
Streptomyces sp. AcE210
Corynebacterium frankenforstense DSM 45800
Streptomyces sp. SID12501
Streptomyces sp. A0958
Streptomyces sp. 4R-3d
Streptomyces niveus NCIMB 11891
Streptomyces sp. SID8354
Streptomyces griseus subsp. griseus JCM 4626
Streptomyces sp. WAC 01529
Streptomyces sp. NL15-2K
Streptomyces sp. cal6
Streptomyces sp. CB03234
Streptomyces sp. SID14515
Streptomyces sp. PTY087I2
Streptomyces sp. Root264
Streptomyces sp. MA5143a
Streptomyces sp. WAC05292
Streptomyces sp. WAC07149
Streptomyces sp. 2132.2
Streptomyces sp. P3
Streptomyces sp. XY332
Streptomyces sp. NRRL S-444
Streptomyces sp. A1547
Rothia sp. HSID18069
Rothia aeria F0184
Rothia sp. HMSC072B03
Rothia sp. HMSC062F03
Rothia sp. HMSC075F09
Rothia sp. HMSC066C03
Rothia mucilaginosa DY-18
Rothia sp. HMSC071B01
Rothia sp. HMSC076D04
Rothia sp. HMSC066H02
Rothia sp. HMSC069C04
Rothia sp. HMSC064F07
Rothia sp. HMSC078H06
Rothia sp. HMSC069D01
Rothia mucilaginosa M508
Rothia sp. HMSC065C12
Rothia sp. HMSC071C12
Rothia sp. HMSC068E02
Actinomadura sp. KC06
Dermacoccus sp. Ellin185
Dermacoccus sp. 147Ba
Frankia sp. CcI49
Streptomyces sp. CB02460
Streptomyces sp. SID7909
Streptomyces sp. CB01249
Kitasatospora sp. CM 4170
Streptomyces sp. CEV 2-1
Streptomyces sp. SID4923
Streptomyces sp. A8642
Yonghaparkia sp. Root332
Streptomyces sp. NRRL F-6122
AccE2
Rhodococcus sp. PBTS 1
Rhodococcus sp. Leaf258
Rhodococcus sp. MEB064
Rhodococcus rhodnii LMG 5362
Schumannella sp. 10F1B-8-1
Rhodococcus sp. 14-2686-1-2
Rhodococcus sp. 86-621-2
Rhodococcus sp. P1Y
Rhodococcus sp. PAMC28705
Rhodococcus sp. 1168
Rhodococcus sp. Eu-32
Rhodococcus sp. 1R11
Rhodococcus sp. 3258
Rhodococcus sp. 05-2256-B3
Rhodococcus sp. 06-235-1A
Rhodococcus sp. 14-2483-1-2
Rhodococcus sp. 05-2254-4
Rhodococcus sp. SBT000017
Rhodococcus sp. 02-925g
Rhodococcus sp. RD6.2
Antrihabitans sp. YC2-6
Nocardia sp. SYP-A9097
Nocardia asteroides NBRC 15531
Nocardia sp. CS682
Nocardia brasiliensis ATCC 700358
Nocardia donostiensis
Nocardia cyriacigeorgica GUH-2
Nocardia sp. FDAARGOS_372
Nocardia farcinica IFM 10152
Rhodococcus sp. SMB37
Rhodococcus sp. BL-253-APC-6A1W
Rhodococcus sp. CX
Rhodococcus sp. DMU1
Rhodococcus rhodochrous KG-21
Rhodococcus sp. BUPNP1
Rhodococcus rhodochrous J45
Rhodococcus pyridinivorans KG-16
Rhodococcus sp. p52
Rhodococcus sp. 2G
Rhodococcus sp. AD45
Rhodococcus sp. OK270
Rhodococcus sp. M1M3W6.2
Rhodococcus sp. IEGM 1241
Rhodococcus erythropolis PR4
Rhodococcus sp. AG1013
Rhodococcus sp. ABRD24
Rhodococcus fascians 1035
Prescottella equi ATCC 33707
Rhodococcus sp. WAY2
Rhodococcus sp. 4CII
Rhodococcus wratislaviensis NBRC 100605
Rhodococcus jostii RHA1
Rhodococcus sp. ACS1
Rhodococcus sp. T7
Rhodococcus sp. NCIMB 12038
Rhodococcus sp. WB9
Rhodococcus sp. LB1
Rhodococcus sp. ACPA1
Skermania sp. ID1734
Pseudonocardia sp. Ae706_Ps2
Microbispora sp. SCL1-1
Microbispora sp. ATCC PTA-5024
Rhodococcus sp. Eu-32
Rhodococcus sp. 14-2686-1-2
Rhodococcus sp. 02-925g
Rhodococcus sp. SBT000017
Rhodococcus sp. 1R11
Rhodococcus sp. 05-2254-4
Rhodococcus sp. 1R11
Rhodococcus sp. 3258
Rhodococcus sp. 05-2256-B3
Rhodococcus sp. WAY2
Rhodococcus sp. T7
Rhodococcus sp. ACS1
Rhodococcus sp. 4CII
Rhodococcus wratislaviensis NBRC 100605
Rhodococcus jostii RHA1
Rhodococcus sp. LB1
Rhodococcus sp. WB9
Rhodococcus sp. ACPA1
Gordonia malaquae NBRC 108250
Williamsia sp. Leaf354
Gordonia otitidis DSM 44809
Gordonia aichiensis NBRC 108223
Gordonia sputi NBRC 100414
Gordonia sp. 852002-50816_SCH5313054-a
Gordonia bronchialis ATCC 25592
Gordonia soli NBRC 108243
Gordonia effusa NBRC 100432
Gordonia sp. SID5947
Gordonia rhizosphera NBRC 16068
Gordonia sp. i37
Gordonia polyisoprenivorans DSM 44266
Gordonia sp. UCD-TK1
Gordonia sp. AC31
Gordonia terrae C-6
Gordonia sp. KTR9
Gordonia sp. YY1
Gordonia sp. 135
Gordonia namibiensis NBRC 108229
Gordonia rubripertincta NBRC 101908
Gordonia alkanivorans NBRC 16433
Gordonia amarae NBRC 15530
Dietzia sp. UCD-THP
Dietzia sp. HMSC21D01
Dietzia sp. IN118
Dietzia sp. ANT_WB102
Corynebacterium sp. KPL1986
Corynebacterium tuberculostearicum SK141
Corynebacterium sp. KPL1821
Corynebacterium lipophiloflavum ATCC 700352
Corynebacterium genitalium ATCC 33030
Corynebacterium appendicis CIP 107643
Corynebacterium sp. NML140438 OX
Corynebacterium sp. NML130628
Corynebacterium sp. HMSC08D02
Corynebacterium sp. HMSC29G08
Corynebacterium sp. HMSC034B08
Corynebacterium sp. HMSC034A01
Corynebacterium sp. NML180780
Corynebacterium sp. HMSC074A01
Corynebacterium sp. HMSC071B10
Corynebacterium sp. NML 150383
Corynebacterium sp. NML 120412
Corynebacterium sphenisci DSM 44792
Corynebacterium frankenforstense DSM 45800
Corynebacterium sp. zg331
Corynebacterium sp. HMSC28B08 OX
Corynebacterium resistens DSM 45100
Corynebacterium sp. KPL1989
Corynebacterium sp. EPI-003-04-2554_SCH2473622
Corynebacterium pyruviciproducens ATCC BAA-1742
Corynebacterium glucuronolyticum ATCC 51866
Corynebacterium doosanense CAU 212
Corynebacterium humireducens NBRC 106098
Corynebacterium marinum DSM 44953
Corynebacterium halotolerans YIM 70093
Corynebacterium cystitidis DSM 20524
Corynebacterium sp. KPL1989
Corynebacterium sp. EPI-003-04-2554_SCH2473622
Corynebacterium sp. J010B-136
Corynebacterium casei UCMA 3821
Corynebacterium sp. LK14
Corynebacterium sp. DNF00584
Corynebacterium sp. HMSC074C01
Corynebacterium aurimucosum ATCC 700975
Corynebacterium sp. HMSC078H07
Corynebacterium sp. HMSC074A09
Corynebacterium sp. HMSC078E09
Gordonia sp. QH-12
Gordonia sihwensis NBRC 108236
Williamsia sp. D3
Williamsia sp. 1138
Williamsia sp. 1135
Corynebacterium kroppenstedtii DSM 44385
Corynebacterium freneyi DNF00450
Corynebacterium lactis RW2-5
Corynebacterium sp. LK12
Corynebacterium sp. ATCC 6931
Corynebacterium maris DSM 45190
Corynebacterium sp. sy039
Corynebacterium sp. TAE3-ERU30
Corynebacterium efficiens DSM 44549
Corynebacterium callunae DSM 20147
Corynebacterium glutamicum ATCC 13032
Corynebacterium deserti GIMN1.010
Corynebacterium argentoratense DSM 44202
Corynebacterium durum F0235
Corynebacterium aquilae DSM 44791
Corynebacterium sp. 13CS0277
Corynebacterium ulcerans 809
Corynebacterium pseudotuberculosis PAT10
Corynebacterium pseudotuberculosis C231
Corynebacterium vitaeruminis DSM 20294
Corynebacterium matruchotii ATCC 14266
Corynebacterium diphtheriae ATCC 700971

Figure S2. Enlarged phylogenetic subtree of Group VI ε-subunit homologs showing the position of AccE2. The enlarged subtree corresponds to Group VI in Fig. 4 and was extracted to better visualize the phylogenetic placement of AccE2. ε-subunit homologs were identified using the PF13822 HMM profile, and the phylogenetic tree was reconstructed based on MAFFT alignment followed by FastTree analysis. Branch support values are shown for selected nodes. AccE2 is marked with a red triangle.
